# The Role of Descending Modulation in Manual Therapy and Its Analgesic Implications: A Narrative Review

**DOI:** 10.1155/2015/292805

**Published:** 2015-12-16

**Authors:** Andrew D. Vigotsky, Ryan P. Bruhns

**Affiliations:** ^1^Kinesiology Program, Arizona State University, Phoenix, AZ 85004, USA; ^2^College of Medicine, University of Arizona, Tucson, AZ 85724, USA

## Abstract

Manual therapy has long been a component of physical rehabilitation programs, especially to treat those in pain. The mechanisms of manual therapy, however, are not fully understood, and it has been suggested that its pain modulatory effects are of neurophysiological origin and may be mediated by the descending modulatory circuit. Therefore, the purpose of this review is to examine the neurophysiological response to different types of manual therapy, in order to better understand the neurophysiological mechanisms behind each therapy's analgesic effects. It is concluded that different forms of manual therapy elicit analgesic effects via different mechanisms, and nearly all therapies appear to be at least partially mediated by descending modulation. Additionally, future avenues of mechanistic research pertaining to manual therapy are discussed.

## 1. Introduction

Manual therapy has been a component of physical rehabilitation programs since as early as 400 BC [1]. Since its inception, many variations of manual therapy techniques have been developed and marketed. Each year, upwards of $8.1 billion is spent in the US on manual therapies, including chiropractic/osteopathic manipulation and massage [2]. Despite the large annual financial expenditures on manual therapies, its mechanisms are not yet fully understood. Current research suggests that a neurophysiological response to manual therapy is responsible for clinically significant decreases in pain [3–8]. Included in the neurophysiological response is the descending pain modulation circuit, which may be a principle mechanism in the analgesic effect of manual therapies.

## 2. Descending Modulation of Pain

Melzack and Wall [9] were the first to explain the potential mechanisms of a central pain modulatory system, wherein the authors described the gate control theory of pain, which simply states that nonnoxious input suppresses painful output by inhibiting dorsal root nociceptors. Gate control is often triggered by touch or nonthreatening sensory input, which activates low-threshold A*β* fibers that inhibit nociceptive input from A*δ* and C afferent fibers [9, 10]. However, another mechanism by which analgesia is induced is through descending modulatory circuits, wherein numerous neurotransmitters, including serotonin (5-HT), vasopressin, oxytocin, adenosine, endocannabinoids, and endogenous opioids (EOs), have been shown to act on structures such as the rostral ventromedial medulla (RVM) and periaqueductal grey (PAG) in order to modulate nociceptive circuits and pain output [11–19]. What is more, and important to consider, is that the analgesic response elicited by human touch [20] and placebo [21–27] is also mediated by EO and endocannabinoids.

*β*-endorphins are EO peptides that have not only been shown to have a comparable analgesic effect to morphine [28], but are 18 to 33 times more potent [29]. Diffuse noxious inhibitory control (DNIC) is the process by which afferent noxious signals are inhibited from the peripheral nervous system (PNS). Using a rat model, Le Bars et al. [30] and Le Bars et al. [31] found that neurons were inhibited by noxious stimuli (a hot bath), therein coining the term DNIC. Since then, multiple studies have suggested that EO is an underlying mechanism of DNIC [32, 33], and more recently it has been suggested that DNIC be referred to as conditioned pain modulation (CPM), by which this mechanism will be referred to herein [34]. It has been suggested that manual therapies that are nociceptive in nature are mediated by CPM [35].

Previous reviews have noted potential descending modulatory mechanisms, an endogenous opioid response, in both physical therapy [36] and physical medicine [37]; however, the neurochemical response to manual therapy and its implications for descending pain modulation, to the authors' knowledge, have not yet been thoroughly reviewed.

## 3. Search Methodology

In order to investigate the neurotransmitters associated with a descending inhibitory response to manual therapy, PubMed was searched using the following query: (“manual therapy” OR “acupressure” OR “neural mobilization” OR “joint manipulation” OR “joint mobilization” OR “massage” OR “manipulative” OR “spinal manipulation”) AND (“vasopressin” OR “naloxone” OR “glial” OR “receptors” OR “biomarkers” OR “cannabinoid” OR “biochemical” OR “endorphin” OR “beta-endorphin” OR “oxytocin” OR “opioid” OR “opioids” OR “serotonin” OR “dopamine” OR “neurotransmitter” OR “neuropeptide” OR “antinociceptive”) NOT (“labor” OR “cardiac” OR “uterus” OR “milk”) in November 2015. All relevant studies and reviews that were written in English were included, with the exception of those that were retracted. Animal studies were included, as they may provide further insight into mechanisms that may not be ethical to directly measure in humans; for example, utilizing brain biopsies to observe receptor activity. The general methods and important findings pertaining to descending pain modulatory systems were described.

## 4. Manipulation Therapies

Through the millennia, numerous types of manipulation therapies have been developed and advocated, and have been purported to cure everything from scarlet fever and diphtheria to hearing loss [1]. However, perhaps the most widely proclaimed outcome from manipulative therapy is pain relief, which may be modulated by neurotransmitters that act on the RVM and PAG.

### 4.1. Osteopathic Manipulative Therapy

Degenhardt et al. [38] recruited twenty male subjects: ten with low back pain and ten without. Four osteopathic manipulative therapy (OMT) techniques (articulatory treatment system, muscle energy, soft tissue technique, and Strain-Counterstrain) were performed on areas of subjects' “somatic dysfunction,” defined as “sites of muscle hypertonicity, tenderness, and joint restriction” [38]. Blood was collected prior to (baseline), 30 minutes, and 24 hours after OMT. Increases in *β*-endorphin and N-palmitoylethanolamide (PEA), an endogenous analog of arachidonylethanolamide (AEA), or anandamide, an endocannabinoid, were observed 30 minutes after treatment; at 24 hours, similar biomarker changes from baseline were found. Subjects with chronic low back pain presented greater biomarker alterations following OMT than the control (asymptomatic) group. However, because no true control or sham group was utilized, it is not possible to distinguish whether these changes in biomarkers were due to the placebo effect or something greater, as endocannabinoids are implicated in placebo-induced analgesia [21], though these data do show that those in pain respond differently to treatment than asymptomatic individuals.

In a blinded, randomized control trial, McPartland et al. [39] investigated the effects of OMT on plasma endocannabinoid concentrations; that is, AEA and 2-arachidonoylglycerol (2-AG). Thirty-one subjects received either an OMT treatment (biodynamic osteopathy in the cranial field) or a sham treatment. Importantly, subjects were recruited from a patient population of an osteopath who regularly uses OMT; therefore, the patients most likely believe the treatment is efficacious. No changes were observed in 2-AG concentrations in either group. In the sham group, negligible, insignificant changes in AEA were observed (17%). The OMT group experienced a 168% increase (5.02 pmol/mL) in AEA over baseline, but this increase did not achieve statistical significance; however, this difference may certainly be clinically relevant, as indicated by changes in Drug Reaction Scale (DRS) scores. These data suggest that endocannabinoids do play a role in the analgesic effect of OMT.

### 4.2. Spinal Manipulation

A number of studies have investigated the pain modulation mechanisms of spinal manipulation, which, as the name implies, is specific only to spinal articulation. The first to do so were Vernon et al. [40], who found a small but statistical increase in plasma *β*-endorphin levels in the experimental group (*n* = 9) who, following a 20-minute relaxation period, underwent a procedure intended to mobilize the upper cervical spine through “joint play maneuvers” [41], during which mild pressure is exerted dorsally on the ligamentous soft tissue of the fixed segment of the neck (the subject is lying supine). Following the introduction of pressure, a fast, low-amplitude rotary thrust is applied that brings the joint through the elastic barrier, producing an “audible or palpable release.” Plasma *β*-endorphin levels were taken at −20, −15, +5, +15, and +30 minutes prior to and following the intervention, and effects were determined via an analysis of variance. Neither the sham group (*n* = 9), which underwent the same joint play manipulation but* without* the thrusting maneuver (only mild, oscillatory pressure was exerted on the fixed segment of the cervical spine while the head and neck underwent passive rotation), nor the control group (*n* = 9) experienced such an increase in plasma *β*-endorphin concentration. However, two subsequent studies demonstrated findings contradicting those of Vernon et al. [40]: Christian et al. [42] and Sanders et al. [43] both failed to find increases in plasma *β*-endorphin concentrations in experimental groups with respect to sham and control groups following spinal manipulation. Sanders et al. [43] drew blood samples −10, +5, and +30 minutes prior to and following the intervention and, like Vernon et al. [40], an analysis of variance was used to examine the presence of a difference of plasma *β*-endorphin levels across all time points. Christian et al. [42] drew three 10 mL blood samples 10 minutes apart prior to the intervention, averaged these values as a baseline, and took 10 mL blood samples five and thirty minutes following the intervention. Of note, Christian et al. [42] described a between-assay coefficient of variation of 11% for 50 pg/tube and 13% for 200 pg/tube plasma *β*-endorphin assays. Relevant methodological differences between the investigations of Christian et al. [42] and Vernon et al. [40] include the division of subjects into four groups by Christian et al. [42]: asymptomatic (*n* = 10) and symptomatic (*n* = 10) groups that received the experimental spinal manipulative therapy (SMT) protocol and asymptomatic (*n* = 10) and symptomatic (*n* = 10) groups that received the sham SMT procedure. Experimental and sham SMT procedures employed by Christian et al. [42] and Vernon et al. [40] were identical. Unlike the studies by Vernon et al. [40] and Sanders et al. [43], no control group was included in that of Christian et al. [42]. Methodological differences in the Sanders et al. [43] study (which included experimental, sham, and control groups each of *n* = 6) from the aforementioned two include the region of the spine considered (the lower lumbar, as opposed to the upper cervical spine), the application of light touch to the affected area in the sham group as opposed to joint play of any kind, and the population sampled. Unlike Christian et al. [42] and Vernon et al. [40], Sanders et al. [43] recruited subjects who were “naïve to chiropractic adjustive manipulation,” as opposed to patients from chiropractic teaching clinics and/or students of the same chiropractic college, which helps to eliminate the potential, previously discussed effect of presuppositions harbored by the sample population. Christian et al. [42] attributed the outcome discrepancies between their data and those of Vernon et al. [40] to between-assay variation, as Vernon et al. [40] reported an 8% increase, which is less than the aforementioned between-assay coefficients of variation.

Recently, Plaza-Manzano et al. [44] compared cervical (*n* = 10) and thoracic manipulations (*n* = 10) to a control group (*n* = 10) in a single-blind, randomized study of graduate student subjects who responded to university-placed advertisements. Cervical manipulations consisted of a high-velocity, mid-range, and leftward rotary thrust about the C4 and C5 vertebrae of the supine subject. Thoracic manipulations consisted of a high-velocity, end-range force applied in the anteroposterior plane to T3-4/T4-5 articulations. Blood was collected from the cephalic vein of each subject before, immediately after, and two hours following the intervention. Both cervical and thoracic groups saw decreases in neurotensin* and* oxytocin, as well as increases in orexin A plasma concentrations following respective interventions.

Multiple reviews have also investigated the pain modulating mechanisms of spinal manipulation [6, 45] and agreed that the analgesic origins are neurophysiological in nature, occurring through some type of descending pain modulation circuit. This is due to observed analgesic effects associated with SMT, including increased pain tolerance and decreased sensitization. The exact circuit, however, is not fully understood, and it appears that different types of spinal manipulations, namely, the velocity with which and the location at which they are performed, may elicit different neurochemical responses indicative of different descending pain modulation mechanisms [46]. For further information on the neurophysiological effects of SMT, readers are directed to Vernon [45] and Pickar [6].

### 4.3. Knee Joint Manipulation

Skyba et al. [47] investigated the effects of knee joint manipulation in rats on monoamine, opioid, and type A *γ*-aminobutyric acid (GABA_A_) receptors in the spinal cord. Knee manipulations employed consisted of the movement of the tibia on the fixed femur. For a duration of three minutes, the joint was flexed and extended across its full range of motion while the tibia was made to translate in the anteroposterior plane. One minute was allowed for rest between each of the three manipulation sessions. Using a model of capsaicin-induced hyperalgesia and the systematic introduction of GABA_A_, opioid, *α*2-adrenergic, 5-HT_1/2_, 5-HT_1A_, 5-HT_2A_, and 5-HT_3_ receptor inhibitors, the authors determined that the analgesic effects of knee joint manipulation were not impacted by the spinal blockade of opioid or GABA_A_ receptors but* were* impacted by the blockade of 5-HT_1A_ and *α*2-adrenergic receptors. It was therefore posited that descending inhibition following knee joint manipulation may be modulated by serotonergic and noradrenergic mechanisms. No attempt has been made to replicate these findings in humans.

## 5. Mobilization Therapies

### 5.1. Ankle Joint Mobilization

There is evidence to support that, in male, Swiss mice, ankle mobilization-induced analgesia is mediated by EO, endocannabinoidergic, and adenosinergic pathways [48–50]. All of these investigations sought to establish the effect of ankle joint mobilization (AJM) on mechanical sensitivity in mice subjected to plantar incision (PI) surgery, for the purpose of inducing an algesic response. AJM was carried out in a manner consistent with Maitland [51]; this involved the rhythmic flexion and extension of the ankle joint following fixation of the knee. The dosage regime of AJM employed is the same regime delineated by Skyba et al. [47], as discussed in the previous section. In the interest of succinctness, the convoluted methods of these investigations and some to follow will not be described in tremendous detail. Readers who are interested in study methodology are directed to each respective study. Each of the studies by Martins and others employed experimental, sham, and control groups. In order to examine the pathways by which analgesia occurred, Martins and others used the appropriate receptor antagonist; for example, naloxone, an opioid receptor antagonist, to determine the role of EO [48]. Mechanical sensitivity following AJM was assessed by measuring the frequency with which mice would withdraw the foot following the application of pressure to its ventral surface. The withdrawal frequency out of 10 pressure applications was taken as a percentage, which constituted each mouse's resultant value.

Importantly, Martins et al. [48] noted that the bottleneck in antihypersensitivity was opioid receptor availability as opposed to opioid-containing leukocytes. Although opioid receptor availability may be the bottleneck in mice, this is not necessarily true for humans. These data should be replicated in human subjects and could have large implications for those in chronic pain or those with central sensitization, as these individuals may have decreased opioid receptor availability [52] and therefore may not benefit as much from this technique.

Further research by Martins et al. [53] investigated the antihyperalgesic and neuroregenerative effects of AJM following a crush injury of the sciatic nerve in adult male Wistar rats. Six groups were studied in order to isolate the effects of AJM so as not to be confounded by the effects of anesthesia and surgery. Mechanical hyperalgesia was tested in a manner similar to the aforementioned studies by Martins and others, and cold hyperalgesia was performed by dropping acetone onto the mid-plantar hind paw. Histological analyses were performed to investigate the effects of AJM on dorsal horn glial cell activation. It was found that glial cell activation and hyperalgesia decreased following AJM. Furthermore, the AJM group had greater myelin sheath thickness. It is possible that these outcomes are related and are of relevance, being that recent research has shown that those in chronic pain exhibit greater glial cell activation [54]; therefore, despite the possibility that AJM may not work well in chronic pain patients through EO-mediated analgesia, it is possible that glial cell inhibition would produce favorable outcomes for chronic pain patients.

### 5.2. Mulligan's Mobilization with Movement

Paungmali and colleagues have studied Mulligan's Mobilization with Movement (MWM) in lateral epicondylalgia [55, 56]. Twenty-four subjects with unilateral chronic lateral epicondylalgia were treated with MWM on six occasions at least two days apart. No statistical decreases in hypoalgesic effects were seen over the treatment period [56]. In a follow-up study, Paungmali et al. [55] failed to antagonize the hypoalgesic effects of MWM with naloxone, an opioid antagonist, and concluded that MWM works through nonopioid methods. However, as noted by Payson and Holloway [57], naloxone by itself can produce an analgesic effect due to its inhibitory effects on inflammation and ischemia; therefore, the results of Paungmali et al. [55] should be called into question.

### 5.3. Neural Mobilization

Neural mobilization (NM) is a type of therapy that purports to relieve adverse neural tension, using methods such as nerve gliding and neural stretching. A systematic review put forth by Ellis and Hing [58] highlights the concerns of methodological quality behind claims of therapeutic efficacy in randomized control trials (RCTs) aimed at studying the clinical utility of neuromanipulative therapy, neurodynamic therapy, or, simply, NM. This review took into account 10 randomized clinical trials (represented by 11 publications), gleaned from the more numerous case studies and other nonblinded trials filling the pool of literature on the topic. Investigative heterogeneity in the 11 studies resulting from dissimilar patient populations considered a variety of pathologies treated and different types of NM techniques analyzed necessitated the use of a qualitative means of assessment rather than meta-analytical means. Ellis and Hing [58] reported that nine of the 11 studies were given, based on the criteria of the PEDro Scale [59], an internal validity score (IVS) of 4 or 5, indicating “moderate” methodological quality. The remaining two studies were given an IVS of 3, indicating “limited” methodological quality. None of the 11 studies satisfied the only two classification items pertaining to subject-therapist blinding. Though a majority of these investigations report a positive clinical benefit from the type of NM employed, Ellis and Hing [58] conclude that only limited evidence exists to support its use. Interestingly, some researchers (such as the next group to be discussed) assert that this noninvasive treatment method has been “*proven* to be clinically effective,” at least in the reduction of pain.

Under the hypothesis that the EO system mediates the reversion of neuropathic pain following neurodynamic treatment and an earlier study suggesting that glial cells and neural growth factor may be involved in the analgesia associated with neural mobilization [60], Santos et al. [61] studied behavioral responses and immunochemical indication of EOs following NM in rats with chronic constriction injury (CCI). CCI, as described by Santos et al. [61], is an induced peripheral nerve injury wherein epineural blood flow (to the sciatic, in this case) is occluded, but not arrested, using chromic gut ligatures. Using male Wistar rats subjected to NM two weeks following CCI (experimental group, *n* = 5) and Western blot assays of the PAG, the authors examined the brains of the rats for *μ*-, *δ*-, and *κ*-opioid receptor expression potentially resulting from the neurodynamic intervention. The NM protocol adopted by the investigators is as follows: rats in the experimental group were anesthetized and placed on their left sides such that the side affected by the CCI (the right) could be manipulated freely; rats in the sham group were just anaesthetized. With the right knee remaining fully extended throughout the session, the investigators flexed the right hip to 70–80° (absolute) until the hamstrings produced a light resistance. At this point, the right ankle was dorsiflexed 30–45° relative to its resting position until a similar resistance (presumably from the gastrocnemius) was detected by the manipulator. Following the establishment of minimal resistance in the manipulated joints, oscillations of the right ankle, wherein the joint was dorsiflexed to 30–45° repeatedly from resting, were initiated. The oscillations were carried out every other day for two minutes, each at 20 oscillations/min with 25-second pauses between them. A total of 10 sessions were completed. Apart from the experimental group (CCI + NM), four other groups were considered. These included “naïve” control, CCI only, sham, and sham + NM groups. Researchers did not find changes in *δ*- or *μ*-opioid receptor expression following the intervention; however, *κ*-opioid receptor expression underwent a significant, 17% increase. These data indicate that the analgesic effects reported by those treated with neural mobilization may be mediated by EOs that act on *κ*-opioid receptors, such as dynorphin A and subtypes thereof.

## 6. Massage Therapies

Massage therapy is often sought for both pleasure and therapy. It has been proposed to work through the gate control theory of pain [62], initially described by Melzack and Wall [9]. However, Field [62] failed to note that different types of massage therapy may work via different mechanisms and did not dive deeply into possible mechanisms. More specifically, light or touch massages may activate low-threshold A*β* fibers, which inhibit nociceptive input from A*δ* and C afferent fibers [9, 10]. Deeper massages, however, may elicit a CPM response due to their pronociceptive nature [35]. Therefore, a more comprehensive review of massage therapy's mechanisms is warranted.

### 6.1. Connective Tissue Massage

Connective tissue massage is intended to both decrease pain and increase range of motion [63]. In the interest of determining the mechanisms behind the pain relief and increases in microcirculation associated with this type of massage, Kaada and Torsteinbø [64] recruited six male and six female subjects, ranging from 23 to 61 years of age, all with a history of “myalgia and various other types of pain.” Of note, the same volunteer cohort was studied in a similar investigation by the pair of authors [65]. Thirty minutes on a bed in a “thermoregulated” room was allowed for each subject to rest prior to the first of four, 9 mL blood samples being taken from the (median) cubital vein. The latter three were taken at 5, 30, and 90 minutes after massage; all were collected in chilled vacutainers containing 0.5 mL of an antiproteolytic buffer. The massage itself was performed by a physiotherapist on laterally positioned, recumbent subjects. Initially, 20 minutes of slow, 1–1.5 in. strokes was applied to the lumbosacral region, including T_12_ and subcostal strokes, giving rise to “sharp, cutting” sensations in the subjects. Following this, 10 minutes was spent treating more local areas of pain specific to each subject using the same stroke length and pressure as applied to the initially treated region. Following the termination of massage treatment and an unspecified storage time on ice (as opposed to conventional, −80°C storage temperatures), blood samples were centrifuged and processed using an assay designed specifically to detect human *β*-endorphin (New England Nuclear *β*-endorphin ^125^I radioimmunoassay). Blood work reportedly indicated a statistical increase in plasma *β*-endorphin levels following connective tissue massage, similar to the* time course* observed in acupuncture and to the* magnitude* observed during exercise. These results are indicative of a CPM response, which modulates pain through descending inhibition.

### 6.2. Acupressure

Using naloxone in male and female Sprague-Dawley rats, Trentini et al. [66] suggested that antinociceptive effects of acupressure applied to areas of low transcutaneous resistance (<30 MΩ), that is, “acupoints,” are mediated by EOs. This conclusion was derived from results obtained from two separate groups of five rats, one of which was subjected to three different trials: (1) an experimental trial in which acupressure was applied to an acupoint, (2) a sham trial in which acupressure was applied to an adjacent point of higher transcutaneous resistance, and (3) a control trial in which no acupressure was applied at all. The other group of five rats, however, was not subjected to a sham trial, but rather to three trials of acupressure at the acupoint 10 minutes after intraperitoneal injection of naloxone (0.05 mg/kg)* or* a saline substitute. No indication of exactly how many of the five rats received naloxone versus saline was provided by the authors. Assessments of antinociception consisted of tail-flick latency tests in which the distal portion of the rats' tails was subjected to a current-containing, tungsten wire heated to 75 ± 5°C. These assessments were performed 10 minutes prior, 10 minutes into, and 20 minutes following the acupressure procedure. Trentini et al. [66] found statistical increases in tail-flick latency during acupressure (10–20 min) and postacupressure (20–40 min) periods in rats injected with* saline* versus those injected with naloxone, indicating possible EO-mediated nociception resulting from acupressure at the acupoint. These results are internally substantiated by the authors' establishment of a statistically greater tail-flick latency in experimental trials as opposed to sham and control trials, which remained at or below baseline for the duration of the assessment.

Despite the findings of Trentini et al. [66], changes in plasma *β*-endorphin levels were not observed in follow-up research in humans [67] following the processing of blood samples collected at baseline, immediately after acupressure treatment, and one hour after acupressure treatment. Like Trentini et al. [66], Fassoulaki et al. [67] only investigated the effects of one experimental acupressure point with respect to one sham acupressure point, but rather than the chosen points being on the hind limb (as was the case in the former), the chosen acupoint and sham point in the latter investigation were both on the face of the human subjects. Thus, the effects of acupressure applied to other parts of the body on *β*-endorphin levels remain unclear. It is also worth noting that both groups of investigators discussed here applied experimental, sham, and control treatments on the same groups of subjects. It was specified by Fassoulaki et al. [67] that each successive treatment for their cohort was performed one day after the last; however, Trentini et al. [66] provided no such specification as to the time allowed between trials in the group of rats studied. From these equivocal data, a potential mechanism by which acupressure may produce analgesia cannot be concluded.

### 6.3. Conventional Massage

Regular massage, consisting of effleurage and other common techniques, has been well studied, but its effects are still not completely understood. Day et al. [68] were the first to note that there is no change in plasma *β*-endorphin or *β*-lipotropin levels following a 30-minute back massage. The possibility of oxytocinergic mechanisms in massage-like stroking in rats was investigated by Agren et al. [69], who tested withdrawal latencies from a hot plate (52°C) in addition to performing the Randall-Selitto test. The rats were injected with saline, oxytocin (1 mg/kg or 0.1 mg/kg), oxytocin antagonist, or a combination of oxytocin and its antagonist (1 mg/kg). The trunk was stroked 50–75 times in 30–45 seconds on both lateral sides, simultaneously, while alternating between ventral and dorsal sides. Withdrawal latency was then retested following massage; investigators reported that the oxytocin antagonist reversed the increases in latency of withdrawal observed in the saline group. Further research in humans (female cyclists) has also demonstrated an oxytocin response to massage [70]. Participants received a 15-minute Swedish massage of the neck and shoulders. Plasma oxytocin levels were measured prior to massage (baseline), during the massage, five minutes following the massage, and 30 minutes following the massage. A nonstatistical increase (Cohen's *d* = 0.89) in plasma oxytocin was observed five minutes after massage, which returned to baseline after 30 minutes. Bello et al. [71] also observed an acute increase in plasma oxytocin in healthy men following 20 minutes of massage applied to the shoulders, upper arms, neck, upper back, and along the spine, but this increase was similar to that observed in the “reading” control at both 15 and 30 minutes following intervention. Additionally, no changes in arginine vasopressin were observed [71]. Similar findings were also observed by Morhenn et al. [72], who compared 15 minutes of Swedish massage on the upper back to rest and found statistical increases in plasma oxytocin and statistical decreases in *β*-endorphin in the massage group relative to the control group. Importantly, a larger sample size was incorporated here, relative to the other studies (experimental: *n* = 65; control: *n* = 30), so the findings of Morhenn et al. [72] may be more reliable and robust when compared to those mentioned previously.

Since then, two studies have found that massage increases urine concentration of dopamine and serotonin [73, 74], suggesting that massage therapy's analgesic effects are mediated by dopaminergic and serotonergic pathways. In addition, a more recent review of the mechanisms of massage therapy noted a 28 and 31% increase in serotonin and dopamine levels, respectively [75].

The longitudinal effects of massage on select catecholamines and neuropeptides have also been investigated. Hart et al. [76] treated young women (mean age = 26) with anorexia nervosa with 30-minute massage twice per week for five weeks. Investigators observed a 72.5% increase in urine dopamine assays, while no statistical change was observed in the control group.

Corticotropin releasing factor (CRF) acts on the locus coeruleus and is associated with analgesic responses, possibly due to the role of the locus coeruleus in modulating ascending and descending pain pathways [82]. Therefore, a CRF response to massage may have analgesic implications. Lund et al. [77] investigated the effects of 30 minutes of massage therapy administered twice weekly for six weeks on urinary CRF concentrations. Increases in urinary CRF concentration were observed following the treatment period, and a nonstatistical increase was observed one month following treatment.

In a randomized-controlled trial in women with breast cancer, subjects received ten twenty-minute effleurage massage treatments over three to four weeks. These treatments were applied to either both feet and lower legs* or* both hands and lower arms. The control group received the same amount of attention but was not touched. Plasma oxytocin levels experienced no statistical changes over the course of therapy in either group; however, a 47.6% decrease in oxytocin in the massage group over the treatment period was observed [78].

Recently, Tsuji et al. [79] carried out a pilot study on the effects of mother-son massage on salivary oxytocin levels in mothers with boys with Autism Spectrum Disorder. After a single 20-minute massage session, no changes in salivary oxytocin concentrations were observed; however, dramatic increases were observed in both the mother and child after massages were given for 20 minutes per day, every day, for three months, when compared to a four-month nonmassage period. It cannot be said for certain whether such outcomes can be generalized to other populations.

Further longitudinal and acute work has been done by Rapaport et al. [81] and Rapaport et al. [80], who investigated the effects of a 45-minute massage or light touch treatment (control) in one session* and* one versus two times per week, for five weeks, on a number of neuroendocrine measures, including arginine vasopressin and oxytocin. In the acute trial, a larger decrease in arginine vasopressin was found relative to the touch group, but no statistical differences were observed for oxytocin [80]. In the longitudinal trial, neuroendocrine samples were collected at 5 minutes and 1 minute prior to the therapy sessions and at 1, 5, 10, 15, 30, and 60 minutes after the end of the session. Two analyses were performed: one that compared baseline (pre) values to the values directly following the final intervention and another that compared baseline (pre) values to the values preceding the final intervention. The latter measure is of particular interest, because it is indicative of longitudinal effects. The following Cohen's* d* effect sizes are presented relative to the touch (control) group. Following the final intervention, the once per week group experienced negligible effects for oxytocin (−0.14). The twice per week group, following the final intervention, noted increased levels of oxytocin (0.50). Preceding the final intervention, negligible increases in oxytocin were noted (0.05) for the once per week massage group. A large effect was observed in the twice per week group for oxytocin (0.92) following the final intervention [81].

From the aforementioned studies, it appears clear that oxytocin plays a role in the analgesic response following conventional massage therapy, but the role of other neuropeptides is unclear.

## 7. Future Research

Being that the analgesic effects of both human touch [20] and placebo [21–27] are mediated by an EO or endocannabinoid response, it is imperative that a placebo control group be utilized in research examining the neurochemical response to manual therapy, as placebo and touch alone are confounding variables. Future research should target therapies that have already been shown to be effective, as to prevent the wasting of resources investigating mechanisms that are not clinically meaningful and should utilize both a control and sham group. Investigators should be cautious when designing experiments that use naloxone, as it can inhibit pain via peripheral mechanisms; thus, it may not be appropriate to use with those who have low back pain [57]. Lastly, it is imperative that researchers be vigilant when interpreting the results of serum levels of EO, as they may not reflect levels seen in the brain or cerebral spinal fluid [83].

## 8. Conclusion

Nearly all types of manual therapy have been shown to elicit a neurophysiological response that is associated with the descending pain modulation circuit; however, it appears that different types of manual therapy work through different mechanisms (Table 1). For example, while massage therapy appears to elicit an oxytocin response, spinal manipulation does not. It is crucial that more higher quality research be performed to better understand these mechanisms, as it can lead to a better understanding of how each therapy can be applied to drive more specific clinical research.

For some therapies, such as manipulation, a minimal amount of force may be required for an analgesic effect [84], but whether a minimum force is required for descending inhibition to occur does not seem to be the case, as touch and placebo alone can trigger a descending inhibitory response. However, this may also be treatment dependent. Being that the gate control theory of pain states that nonnoxious stimuli inhibit noxious stimuli, more aggressive therapies may be too noxious to trigger a gate control response, but not noxious enough to produce a CPM response. Thus, more research is needed to shed light on these paradoxical treatment outcomes.

Despite the large popularity and long history of manual therapy, its mechanisms are not truly understood. Understanding these mechanisms may help researchers and clinicians to choose which therapy is most appropriate for each patient or subpopulation and may also lead to more effective therapies in the future.

## Figures and Tables

**Table 1:** Neurophysiological response to manual therapy variations.

Study	Subjects	Control/sham	Variation	Findings
Degenhardt et al. [38]	7 women and 3 men with (*n* = 10) and without (*n* = 10) low back pain	Light touch	OMT	↑ *β*-endorphins ↑ PEA

McPartland et al. [39]	Osteopathic patient population (*n* = 31)	Sham manipulation	OMT	↑ AEA

Vernon et al. [40]	27 healthy males	(1) Control group laid supine on a treatment table (2) Sham group received joint play maneuvers	SMT	↑ *β*-endorphins

Christian et al. [42]	40 male subjects who were chiropractic patients and students with and without pain	Sham (joint taken to end-range of motion)	SMT	→ *β*-endorphins

Sanders et al. [43]	9 males and 9 females with acute (<2 weeks) low back pain	Sham group (*n* = 6) received light touch at L4/L5–S1	SMT	→ *β*-endorphins

Plaza-Manzano et al. [44]	30 graduate school students	No treatment	SMT	↑ orexin A ↓ neurotensin ↓ oxytocin

Skyba et al. [47]	113 male Sprague-Dawley rats	(1) Vehicle w/manipulation (2) Vehicle w/anesthesia (3) Drugs w/anesthesia	Knee manipulation	Serotonin-mediated Norepinephrine-mediated Non-GABA-mediated

Martins et al. [48]	8 male Swiss mice per group	(1) Control (2) Sham	Ankle joint mobilization	EO-mediated^†^

Martins et al. [49]	8 male Swiss mice per group	(1) Control (2) Sham	Ankle joint mobilization	CBR-mediated^†^

Martins et al. [50]	8 male Swiss mice per group	(1) Control (2) Sham	Ankle joint mobilization	Adenosine-mediated^†^

Martins et al. [53]	8 adult male Wistar rats per group	(1) Sham (2) Sham w/anesthesia (3) Sham w/mobilization (4) Crush (5) Crush w/anesthesia	Ankle joint mobilization	↓ glial cell activation

Paungmali et al. [56]	7 females and 17 males with lateral epicondylalgia	(1) Placebo (2) Control	MWM	No increase in tolerance over treatment period

Paungmali et al. [55]	4 females and 14 males with lateral epicondylalgia	(1) Placebo (2) Control	MWM	Non-EO-mediated^†^

Santos et al. [61]	Male Wistar rats	(1) Control (2) Injury only (3) Sham (4) Sham w/mobilization	NM	Dynorphin-mediated

Kaada and Torsteinbø [64]	6 male and 6 female subjects with a history of myalgia		Connective tissue massage	↑ *β*-endorphins

Trentini et al. [66]	Male and female Sprague-Dawley rats	(1) Control (2) Placebo	Acupressure	EO-mediated^†^

Fassoulaki et al. [67]	4 females and 8 males without a familiarity with acupuncture	(1) Control (2) Sham	Acupressure	→ *β*-endorphins

Day et al. [68]	17 women and 14 men who were healthy and free of pain	Control	Conventional massage	→ *β*-endorphins → *β*-lipotropins

Agren et al. [69]	13–21 male Sprague-Dawley rats	Control	Conventional massage	Oxytocin-mediated^†^

Turner et al. [70]	26 nulliparous women that cycle	(1) Positive emotion (2) Negative emotion	Conventional massage	↑ oxytocin

Bello et al. [71]	14 males	Control	Conventional massage	↑ oxytocin → arginine vasopressin

Morhenn et al. [72]	50 females and 45 males	Rest	Conventional massage	↑ oxytocin ↓ *β*-endorphins

Hernandez-Reif et al. [73]	13 women and 11 men with >6 months low back pain	Relaxation therapy	Conventional massage	↑ dopamine ↑ serotonin

Hernandez-Reif et al. [74]	34 women with stage 1 or 2 breast cancer	Control (medical treatment only)	Conventional massage	↑ dopamine ↑ serotonin

Field et al. [75]^*∗*^	Review		Conventional massage	↑ dopamine ↑ serotonin

Hart et al. [76]	Nineteen women with anorexia nervosa	Control (standard treatment only)	Conventional massage	↑ dopamine

Lund et al. [77]	19 fibromyalgia patients	Guided relaxation	Conventional massage	↑ corticotropin releasing factor

Billhult et al. [78]	32 women with breast cancer	Attention	Conventional massage	↑ oxytocin

Tsuji et al. [79]	7 Japanese boys with Autism Spectrum Disorder and their mothers	Control (no massage, crossover)	Conventional massage	↑ oxytocin

Rapaport et al. [80]	29 females and 24 males	Light touch	Conventional massage	→ oxytocin ↓ arginine vasopressin

Rapaport et al. [81]	23 females and 22 males	Light touch	Conventional massage	↑ oxytocin (acute) → oxytocin (chronic)

*∗* denotes review; † denotes a conclusion inferred from naloxone or relevant antagonistic response.
