# Corrigendum to “The Role of Descending Modulation in Manual Therapy and Its Analgesic Implications: A Narrative Review”

**DOI:** 10.1155/2017/1535473

**Published:** 2017-10-26

**Authors:** Andrew D. Vigotsky, Ryan P. Bruhns

**Affiliations:** ^1^Kinesiology Program, Arizona State University, Phoenix, AZ 85004, USA; ^2^College of Medicine, University of Arizona, Tucson, AZ 85724, USA

In the article titled “The Role of Descending Modulation in Manual Therapy and Its Analgesic Implications: A Narrative Review” [1], there was an error in the interpretation of the data presented by Plaza-Manzano et al. (2014). Therefore, in the Spinal Manipulation section, the sentence “Both cervical and thoracic groups saw decreases in neurotensin and oxytocin, as well as increases in orexin A plasma concentrations following respective interventions” should be corrected to “Both cervical and thoracic groups saw increases in neurotensin and oxytocin, as well as decreases in orexin A plasma concentrations following respective interventions.”

Additionally, in the Conclusion section, the text reading “Nearly all types of manual therapy have been shown to elicit a neurophysiological response that is associated with the descending pain modulation circuit; however, it appears that different types of manual therapy work through different mechanisms (Table 1). For example, while massage therapy appears to elicit an oxytocin response, spinal manipulation does not. It is crucial that more higher quality research be performed to better understand these mechanisms, as it can lead to a better understanding of how each therapy can be applied to drive more specific clinical research” should be corrected as follows.

“Nearly all types of manual therapy have been shown to elicit a neurophysiological response that is associated with the descending pain modulation circuit; however, it appears that potential analgesic responses to different types of manual therapy may be modulated by different mechanisms (Table 1). For example, while osteopathic manipulative therapy appears to elicit a *β*-endorphin response, conventional massage does not. It is crucial that higher quality research with better controls be carried out to better understand potential mechanisms, as this will lead to a better understanding of how each therapy may be applied to drive more specific clinical research.”

Finally, Table 1 should be corrected as follows.

## Figures and Tables

**Table 1:** Neurophysiological response to manual therapy variations.

Study	Subjects	Control/sham	Variation	Findings
Degenhardt et al. [38]	7 women and 3 men with (*n* = 10) and without (*n* = 10) low back pain	Light-touch	OMT	↑ *β*-endorphins ↑ PEA

McPartland et al. [39]	Osteopathic patient population (*n* = 31)	Sham manipulation	OMT	↑ AEA

Vernon et al. [40]	27 healthy males	(1) Control group laid supine on a treatment table (2) Sham group received joint play maneuvers	SMT	↑ *β*-endorphins

Christian et al. [42]	40 male subjects who were chiropractic patients and students with and without pain	Sham (joint taken to end-range of motion)	SMT	→ *β*-endorphins

Sanders et al. [43]	9 males and 9 females with acute (<2 weeks) low back pain	Sham group (*n* = 6) received light touch at L4/L5–S1	SMT	→ *β*-endorphins

Plaza-Manzano et al. [44]	30 graduate school students	No treatment	SMT	↓ *orexin A* ↑ *neurotensin* ↑ *oxytocin*

Skyba et al. [47]	113 male Sprague-Dawley rats	(1) Vehicle w/manipulation (2) Vehicle w/anesthesia (3) Drugs w/anesthesia	Knee manipulation	Serotonin-mediated Norepinephrine-mediated Non-GABA-mediated

Martins et al. [48]	8 male Swiss mice per group	(1) Control (2) Sham	Ankle joint mobilization	EO-mediated^†^

Martins et al. [49]	8 male Swiss mice per group	(1) Control (2) Sham	Ankle joint mobilization	CBR-mediated^†^

Martins et al. [50]	8 male Swiss mice per group	(1) Control (2) Sham	Ankle joint mobilization	Adenosine-mediated^†^

Martins et al. [53]	8 adult male Wistar rats per group	(1) Sham (2) Sham w/anesthesia (3) Sham w/mobilization (4) Crush (5) Crush w/anesthesia	Ankle joint mobilization	↓ glial cell activation

Paungmali et al. [56]	7 female and 17 males with lateral epicondylalgia	(1) Placebo (2) Control	MWM	No increase in tolerance over treatment period

Paungmali et al. [55]	4 female and 14 males with lateral epicondylalgia	(1) Placebo (2) Control	MWM	Non-EO-mediated^†^

Santos et al. [61]	Male Wistar rats	(1) Control (2) Injury only (3) Sham (4) Sham w/mobilization	NM	Dynorphin-mediated

Kaada and Torsteinbø [64]	6 male and 6 female subjects with a history of myalgia		Connective tissue massage	↑ *β*-endorphins

Trentini et al. [66]	Male and female Sprague-Dawley rats	(1) Control (2) Placebo	Acupressure	EO-mediated^†^

Fassoulaki et al. [67]	4 females and 8 males without a familiarity with acupuncture	(1) Control (2) Sham	Acupressure	→ *β*-endorphins

Day et al. [68]	17 women and 14 men who were healthy and free of pain	Control	Conventional massage	→ *β*-endorphins → *β*-lipotropins

Agren et al. [69]	13–21 Male Sprague-Dawley rats	Control	Conventional Massage	Oxytocin-mediated^†^

Turner et al. [70]	26 nulliparous women that cycle	(1) Positive emotion (2) Negative emotion	Conventional massage	↑ oxytocin

Bello et al. [71]	14 males	Control	Conventional massage	↑ oxytocin → arginine vasopressin

Morhenn et al. [72]	50 females and 45 males	Rest	Conventional massage	↑ oxytocin ↓ *β*-endorphins

Hernandez-Reif et al. [73]	13 women and 11 men with >6 months low back pain	Relaxation therapy	Conventional massage	↑ dopamine ↑ serotonin

Hernandez-Reif et al. [74]	34 women with stage 1 or 2 breast cancer	Control (medical treatment-only)	Conventional massage	↑ dopamine ↑ serotonin

Field et al. [75]^*∗*^	Review		Conventional massage	↑ dopamine ↑ serotonin

Hart et al. [76]	Nineteen women with anorexia nervosa	Control (standard treatment-only)	Conventional massage	↑ dopamine

Lund et al. [77]	19 fibromyalgia patients	Guided relaxation	Conventional massage	↑ corticotropin releasing factor

Billhult et al. [78]	32 women with breast cancer	Attention	Conventional massage	↑ oxytocin

Tsuji et al. [79]	7 Japanese boys with Autism Spectrum Disorder and their mothers	Control (no massage, crossover)	Conventional massage	↑ oxytocin

Rapaport et al. [80]	29 females and 24 males	Light touch	Conventional massage	→ oxytocin ↓ arginine vasopressin

Rapaport et al. [81]	23 females and 22 males	Light touch	Conventional massage	↑ oxytocin (acute) → oxytocin (chronic)

*∗*denotes review; †denotes a conclusion inferred from naloxone or relevant antagonistic response.
